# Optical Genomic Mapping Identified a Heterozygous Structural Variant in *NCF2* Related to Chronic Granulomatous Disease

**DOI:** 10.1007/s10875-022-01331-4

**Published:** 2022-07-28

**Authors:** Xiaoying Hui, Jingmin Yang, Jing Zhang, Jinqiao Sun, Xiaochuan Wang

**Affiliations:** 1 Department of Allergy and Clinical Immunology, Children’s Hospital of Fudan University, 399 Wanyuan Road, Shanghai, 201102 China; 2 Key Laboratory of Birth Defects and Reproductive Health of National Health and Family Planning Commission (Chongqing Key Laboratory of Birth Defects and Reproductive Health, Chongqing Population and Family Planning, Science and Technology Research Institute), Chongqing, 400020 China; 3 State Key Laboratory of Genetic Engineering, School of Life Sciences, Fudan University, Shanghai, China; 4 Shanghai WeHealth Biomedical Technology Co., Ltd., Shanghai, China; 5 Shanghai Institute of Infectious Disease and Biosecurity, Shanghai, 200032 China

To the editor

Chronic granulomatous disease (CGD) is an inherited rare immunodeficiency characterized by defects of superoxide production by phagocytic nicotinamide adenine dinucleotide phosphate (NADPH) oxidase [1]. This enzyme is composed of five subunits, encoded by *CYBB*, *CYBA*, *NCF1*, *NCF2*, and *NCF4*. NCF2-related CGD is autosomal recessively inherited, and worldwide accounts for ~ 5% of all cases. Pathogenic variants of *NCF2* were previously thought to be mainly single-nucleotide variants (SNVs) located on exons or their nearby splicing sites. Hence, whole exome sequencing (WES) is widely used for genetic testing of CGD in our center, yet sometimes fail or only find one variant. Copy number variants (CNVs) or structural variation (SVs) were sometimes the answer. So far, only three cases of *NCF2*-CGD with homozygous SVs have been reported: one is ~ 1.1-kb duplication [2], two deletions with one exact size unknown [3], and the other 1,380 bp [4]. All these based on reverse transcript polymerase chain reaction (RT-PCR). Yet samples for RNA extraction were not always available and thus not convenient than DNA sequencing. And a short turnaround time (TAT) is also anticipated for patients to access timely therapy. Comparative genomic hybridization (CGH) has a short TAT, yet with a minimum size of 30 kb; to our knowledge, large SVs are rare in CGD-related genes, except few reported in *CYBB*. Re-analysis of WES data also seems fast, which was hindered by unavailable raw data. Genome-wide long-read sequencing is too expensive. Taken all into consideration, a simplified and fast cytogenetic technology, optical genomic mapping (OGM), was welcomed. This study reports the utility of OGM to identify the first heterozygous SV deletion of NCF2-CGD (P1).

P1 is a 12-year-old female with onset of CGD in the neonatal period. She manifested with recurrent lift-threatening infections of bacteria, fungus, mycobacteria, and excessive inflammatory responses leading to pulmonary granuloma formation. The infections affect multiple organs, including lung, lymph node, skin, abdominal cavity, and central nervous system. She was diagnosed with bacillus Calmette-Guérin infection, lymphatic tuberculosis, tuberculosis, and abdominal tuberculosis at the age of 3 years, and recovered from anti-tuberculosis treatment at the age of 5 years. Defective respiratory burst was detected by dihydrorhodamine-1,2,3 (DHR) test, and it showed a stimulation index value of 2.3 (normal range > 100; Fig. 1a). The gp91-phox subunit of NADPH oxidase (encoded by *CYBB*) was normal according to flow cytometry–based extracellular staining with Moab 7D5. WES previously identified a heterozygous variant NM_000433.4:c.1130_1135 del in *NCF2* (Fig. 1b). Sanger sequencing confirmed its maternal origin (Fig. 1b). This variant is predicted to lead to an in-frame deletion of two amino acids (p.D377_M378del), which may not lead to protein degradation but affect the PB1 domain (351–429 aa). As known, the PB1 domain is vital for interaction of NCF2 to other cytoplasmic components of NADPH oxidase (NCF4 and NCF1). Disturbing this interaction inhibits NCF2’s translocation to membrane to assemble with CYBB and CYBA to regulate electron transfer, and thus impaired activating of superoxide production (Supplementary Methods and Results). Suspecting an SV or CNV may be the second pathogenic variant for *NCF2*; OGM was further applied.

**Fig. 1:**
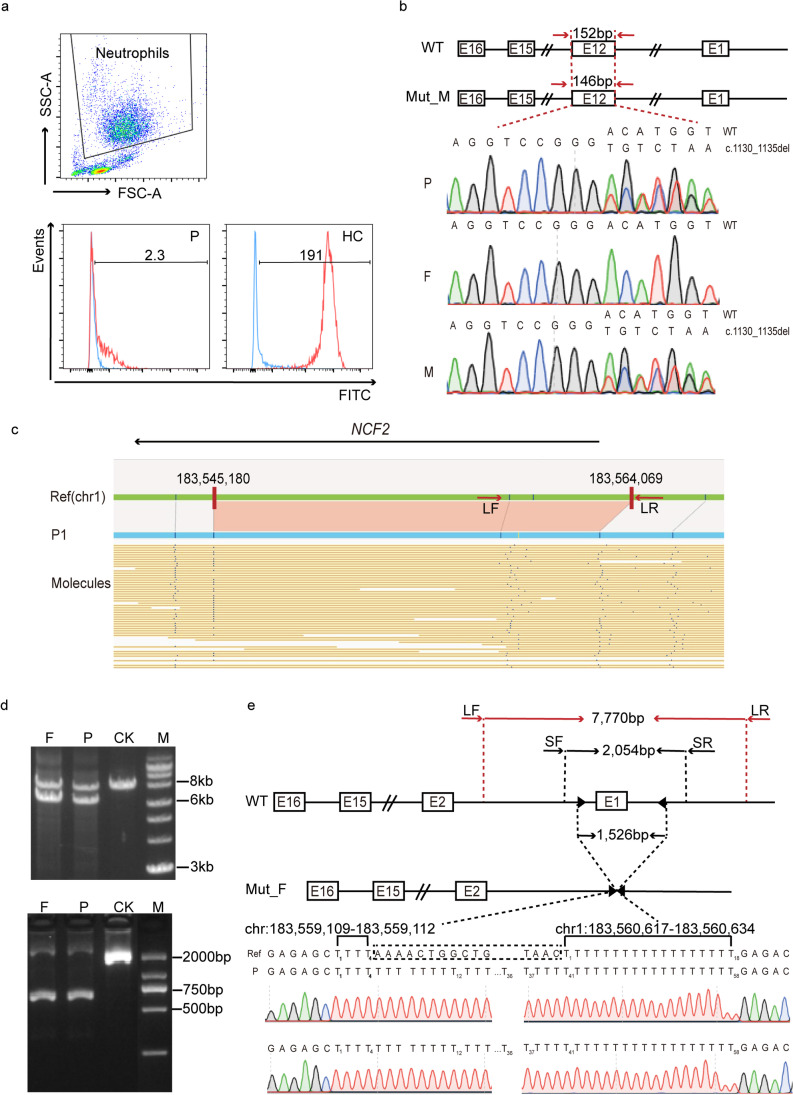
*NCF2* biallelic pathogenic variants identified in a patient diagnosed with chronic granulomatous disease*.* **a** DHR-1,2,3 assay results for P1. The upper image shows a flow cytometry dot plot of whole blood specimen (red blood cells were lysed). The neutrophil population has been gated. FSC, forward scatter; SSC, side scatter. The lower image shows the results of DHR analysis after stimulation with phorbol-12-myrismte-14-acetate (PMA), for this CGD patient (P) and a healthy donor (HC). Blue line indicates the unstimulated condition; red line represents the PMA-stimulated condition. The number in each figure indicates the stimulation index. FITC, fluorescein isothiocyanate. **b** A heterozygous *NCF2* variant (NM_000433:c.1130_1135del) identified in P1 by exome sequencing. Sanger sequencing confirmed a heterozygous variant c.1130_1135 del and its maternal origin. This variant resulted in a 6-bp shorter exon 12 (146 bp). WT, wildtype; Mut_M, mother. F, father. **c**. A 1457-bp heterozygous deletion on NCF2 was identified by optical genomic mapping (shown is a genome browser view). Ref, GRCh37/hg19 reference sequence of chromosome 1 (Chr1), also known as NC_000001.10. One molecule stands for one constructed genome map, and its pattern of labels alignment to reference indicates the deletion and heterozygosity. **d** Results of agarose electrophoresis for PCR products amplified by long-range PCR (upper, 1% agarose) and following short range PCR (lower, 3% agarose). Lower bands indicating the deletion were subjected to sequencing. P, patient; F, father; CK, healthy control; M, DNA ladder; kb, kilobase; b, base pair. **e** An overview of breakpoint identification. The upstream and downstream breakpoints were found among chr1: 183,559,109–183,559,112 and chr1:183,560,617–183,560,634, respectively. Note that both sites were thymine rich on references (4 and 18, individually). WT, wild-type genome sequencing of *NCF2*; E, exons; LF, LR, SF, and SR are primer pairs designed for long-range (L) and short-range (S) PCR. Mut_F, mutated sequencing of *NCF2* from P1’s father

OGM utilizes to image ultra-long, megabase-size linear DNA molecules that are fluorescently labeled specifically at CTTAAG motifs, to detect genome-wide SVs in a high resolution in one assay with a TAT of 1 week. Labels are distributed at 14 ~ 17 per 100-kb genome and totally ~ 500,000 throughout the genome. Label positions linked by a gray line demonstrate the alignment between reference and a sample (Fig. 1c; Figure S1). Changes in label spacing, order, position, and orientation of the label patterns show major SV types: deletion/insertion (> 500 bp), inversion (> 30 kb), duplication (> 30 kb), and translocation (> 50 kb) (Figure S1). OGM offers 95% sensitivity for heterozygous insertions/deletions > 500 bp (99% for homozygous). Variants missed by CGH or short-read sequencing (such as tandem repeats, inversions, or translocations) may also detectable. Besides, an internal SV control database of healthy people favors variants filtering down and identification of disease-related variations. Of note, OGM is imaging not sequencing, and the size/location it provided is approximately calculated. Thus, further confirmation by Sanger sequencing is necessary.

OGM was performed as described with modifications [5]. DNA extraction, labeling, quality control, de novo assembly, and SV calling are detailed in Supplementary Methods and Results. SVs detected in P1 (before/after filtering) were used as examples (Figures S1, S2). OGM detected totally 6795 CNVs/SVs in P1: including 4570 insertions, 1913 deletions, 146 inversions, 45 duplications, 63 translocations, 18 CNV gain segments, and 40 CNV loss segments as represented in a Circos plot (Figure S2). SVs filtered out were (1) with low confidence (cutoff value was recommended by Bionano), (2) detected in healthy control database, (3) did not overlap with curated genes (namely, CGD genes). Finally, only one left a heterozygous *NCF2* deletion (Figure S2). From a genome browser view (Fig. 1c), this 1457-bp deletion was embedded on chr1:183,545,180–183,564,069, as indicated by a shorter distance and one label lost (ref: four labels) between sample and reference (17,432 bp vs 18,889 bp; Fig. 1c). To further investigate the breakpoints of this deletion, chr1:183,557,412–183,565,181 (length 7770 bp, with the speculated deletion) were first amplified using LongAmp Taq PCR Kit (E5200S; NEB, Ipswich, MA). Primer pairs and PCR conditions were LF: 5′-TAAAAAAGATAGGGAAATAGGAGAAGC-3′, and LR: 5′-TGAACACACATTAGGAGAGGTAGAGAG-3′; 96 °C for 5 min followed by 35 cycles of 96 °C for 30 s, 60 °C for 30 s, and 72 °C for 4 min, with a final extension at 72 °C for 4 min. A predicted 6314 bp stands for the deleted variant (Fig. 1d, upper; Table S1). The short band was subjected to targeted next-generation sequencing (Targeted-NGS, as routinely operated for WES, Supplementary Methods and Results). The targeted range was narrowed down to chr1: 183,558,933–183,560,986 (length 2054 bp; Fig. 1d, lower; Table S1). And it was amplified using primer pairs (SF: 5′-TGTAAAGCGCTGGGATAGATAGAG-3′ and SR: 5′-ATTTTGACATTTGCAGGAAATTGG-3′). The deletion resulted in a 598-bp band. Sanger sequencing identified the deletion size (1506 ~ 1526 bp), breakpoints, and its paternal origin (Fig. 1e). The upstream break was among chr1: 183,559,109–183,559,112 (four thymines in wild type), and downstream within chr1: 183,560,617–183,560,634 (18 thymines in wild type), respectively. This deletion involves the 5′-untranslated region (5′-UTR), exon 1, and partial intron 1. Loss of exon 1, where translation of *NCF2* initiates, is predicted to a complete loss of NCF2 synthesis (NP_000424.2). The limitation of this study is that detection of the NCF2 protein due to this SV was technically unsuccessful. Samples for obtaining P1’s RNA to confirm deficient *NCF2* expression was also restricted by the COVID-19 pandemic.

In summary, a heterozygous pathogenic *NCF2* variant was identified by OGM, and breakpoints were confirmed by long-range PCR and Sanger sequencing. Together with the SNV detected by WES, P1 is finally diagnosed both clinically and genetically. This case represents the first use of OGM to solve an undiagnosed heterozygous case within the *NCF2*-CGD. These discoveries warrant a necessary of considering SVs for CGD, and OGM is sometimes helpful especially when the variant is estimated at a range of 500 bp ~ 1 Mb.

## Supplementary Information

Below is the link to the electronic supplementary material.

Supplementary file1 (PNG 837 KB)

Supplementary file2 (TIF 64937 KB)

Supplementary file3 (PNG 810 KB)

Supplementary file4 (TIF 54090 KB)

Supplementary file5 (DOCX 23 KB)

Supplementary file6 (DOCX 33 KB)

## Data Availability

Regarding data and privacy protection, the dataset supporting the conclusions of the article is available upon individual request directed to the corresponding author.
