# Supplementary figures and images for: Optical Genomic Mapping Identified a Heterozygous Structural Variant in NCF2 Related to Chronic Granulomatous Disease
Source: J Clin Immunol. 2022 Jul 28;42(8):1614–7. doi: 10.1007/s10875-022-01331-4 (PMC9330964; doi:10.1007/s10875-022-01331-4)

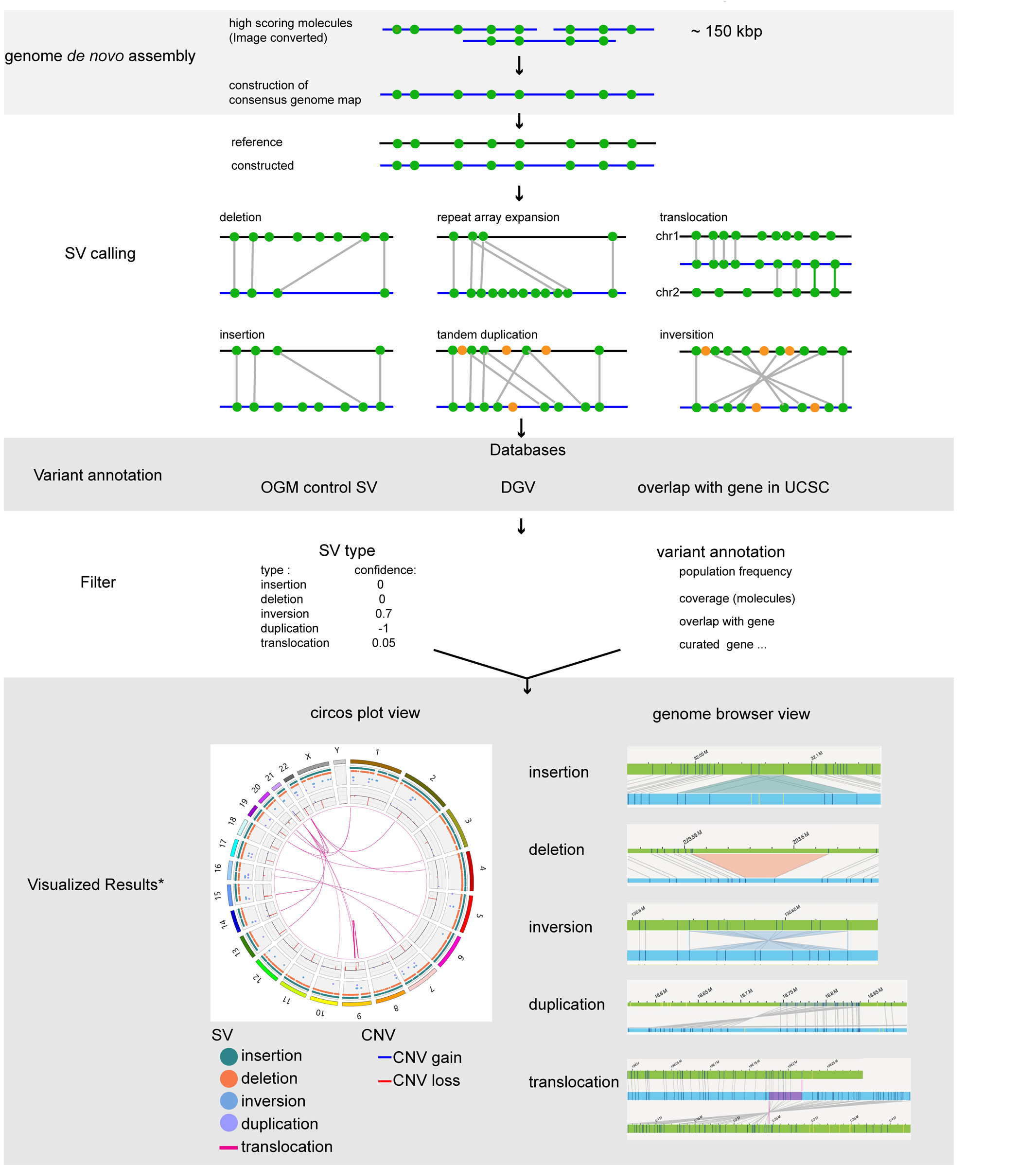

Supplement: Supplementary file 1 — Supplementary file1 (PNG 837 KB) [file 10875_2022_1331_MOESM1_ESM.png]

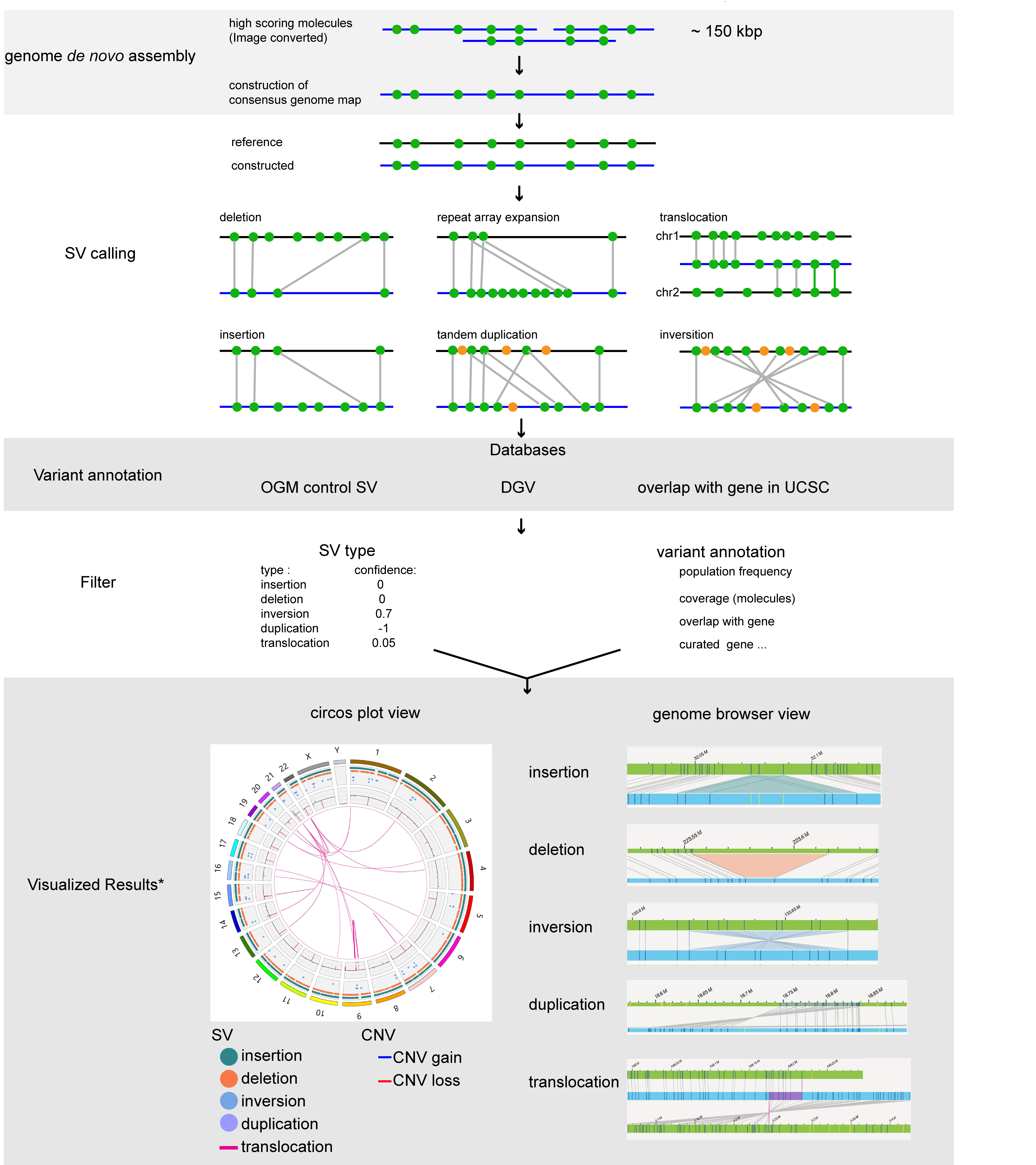

Supplement: Supplementary file 2 — Supplementary file2 (TIF 64937 KB) [file 10875_2022_1331_MOESM2_ESM.tif]

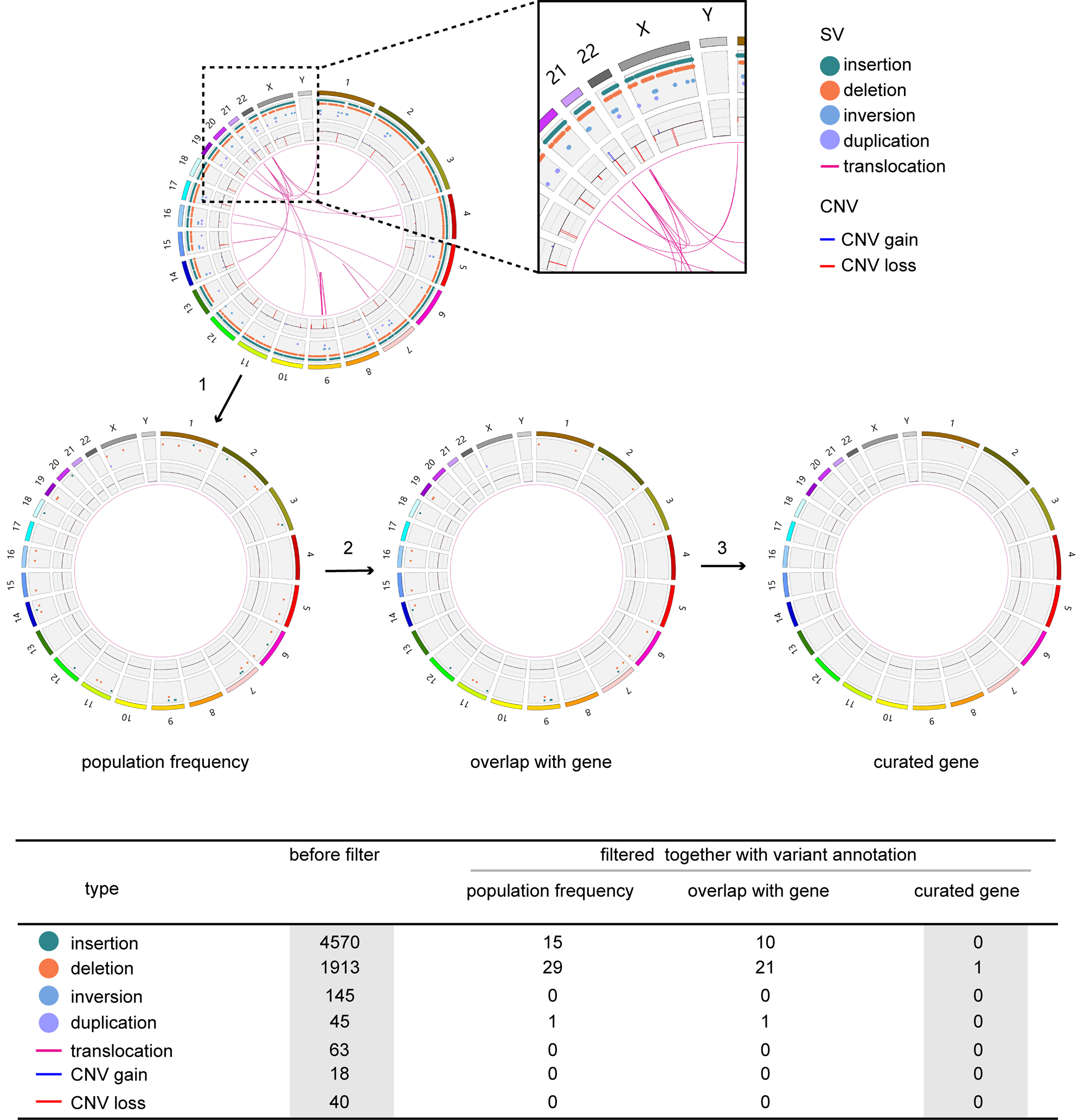

Supplement: Supplementary file 3 — Supplementary file3 (PNG 810 KB) [file 10875_2022_1331_MOESM3_ESM.png]

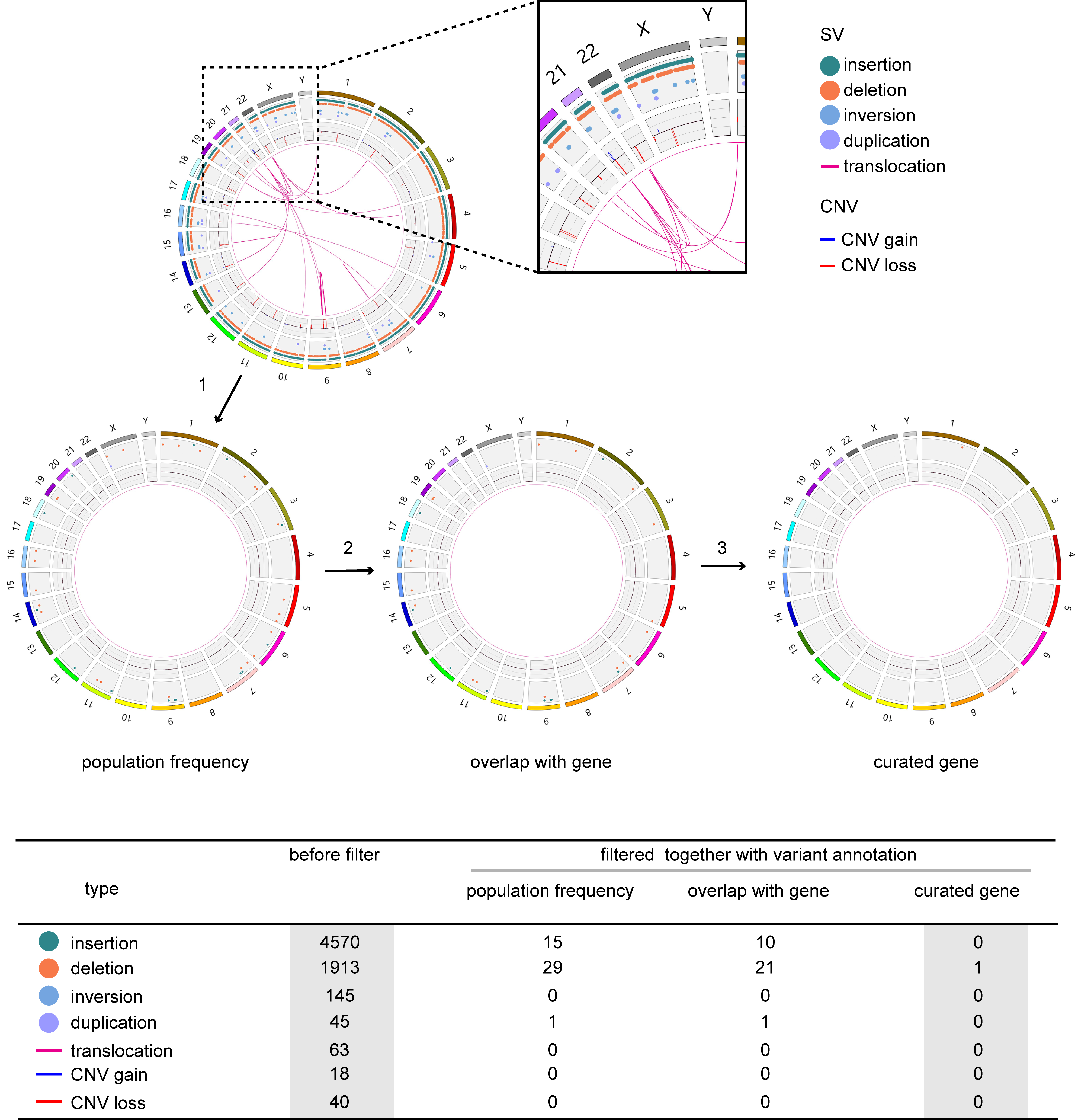

Supplement: Supplementary file 4 — Supplementary file4 (TIF 54090 KB) [file 10875_2022_1331_MOESM4_ESM.tif]
