# Supplementary material for: Optical Genomic Mapping Identified a Heterozygous Structural Variant in NCF2 Related to Chronic Granulomatous Disease
Source: J Clin Immunol. 2022 Jul 28;42(8):1614–7. doi: 10.1007/s10875-022-01331-4 (PMC9330964; doi:10.1007/s10875-022-01331-4)
Supplement: Supplementary file 5 — Supplementary file5 (DOCX 23 KB) [file 10875_2022_1331_MOESM5_ESM.docx]

| Table S1. Primers used for targeted-long PCR and Sanger sequencing | | | | | |
| --- | --- | --- | --- | --- | --- |
|  | Amplified region | Sequence of oligonucleotide（5’-3’） | | Length predicted  (control / patient) | |
| Long-PCR | chr1:183,557,412-183,565,181 | LF | TAAAAAAGATAGGGAAATAGGAGAAGC | 7770 | 6314 |
|  |  | LR | TGAACACACATTAGGAGAGGTAGAGAG |  |  |
| Sanger sequencing | chr1:183,558,933-183,560,986 | SF | TGTAAAGCGCTGGGATAGATAGAG | 2054 | 598 |
|  |  | SR | ATTTTGACATTTGCAGGAAATTGG |  |  |
