# Supplementary material for: Optical Genomic Mapping Identified a Heterozygous Structural Variant in NCF2 Related to Chronic Granulomatous Disease
Source: J Clin Immunol. 2022 Jul 28;42(8):1614–7. doi: 10.1007/s10875-022-01331-4 (PMC9330964; doi:10.1007/s10875-022-01331-4)
Supplement: Supplementary file 6 — Supplementary file6 (DOCX 33 KB) [file 10875_2022_1331_MOESM6_ESM.docx]

**Supplementary Methods and Results**

**Methods**

**1.1 Measurement of NADPH Oxidase Activity in Neutrophils by DHR assay**

DHR test was performed as described (1). In brief, whole blood samples were incubated with DHR and stimulated with phorbol-12-myrismte-14-acetate (PMA) for 20 min. Then, red blood cells were lysed with hemolysin. After stimulation and lysis, samples were analyzed using a FACSCalibur flow cytometer (Becton Dickinson, Franklin Lakes, NJ, USA). PMA activates the neutrophil oxidase to produce reactive oxygen intermediates(ROI) especially hydrogen peroxide (H_2_O_2_). H_2_O_2_ oxidizes the non-fluorescent DHR into fluorescent rhodamine 123 which can be measured via flow cytometry in the fluorescein isothiocyanate channel (at excitation wavelength of 488nm). Normal bloods produce a strong fluorescence, whereas patients with an abnormality in ROI production produce a weak level or no fluorescence at all. Stimulation index (SI) was defined as geometric mean of fluorescence intensity of PMA-inbubated neutrophils / geometric mean of fluorescence intensity of PBS-incubated neutrophils.

**1.2 Whole-exome sequencing**

Genomic DNA(gDNA) was extracted from ethylenediaminetetraacetic acid (EDTA)-treated peripheral blood using QIAamp DNA Blood Mini Kit (51106; Qiagen, Germany). gDNA was enriched with the xGen Exome Research Panel v2.0 (Integrated DNA Technologies, Coralville, Iowa) and sequenced on HiSeq 2000/2500 (Illumina, San Diego, CA) to generate 150 bp paired end reads at a target depth of 100 ×. Average sequencing coverage was >=100-fold with >96% of the region-of-interest covered at least 20-fold. The quality of raw data was checked by FASTQC (https://www.bioinformatics.babraham.ac.uk/projects/fastqc/). After removing the low-quality reads, the adaptor reads were mapped to the reference genome (GRCh37/hg19) with Burrows-Wheeler Aligner (BWA). GATK was used for insertion and deletion realignment, quality recalibration, and variant calling. Detected variants were annotated using ANNOVAR. Variants with minor allele frequencies (MAFs) of >0.5% were filtered based on frequency in healthy population databases including 1000 Genome Project (1000G, http://www.internationalgenome.org/data), the Exome Aggregation Consortium Browser (ExAC, http://exac.broadinstitute.org), and the Genome Aggregation Database (gnomAD, https://gnomad.broadinstitute.org/). Novel and rare variants (MAF<0.5%) were classified and analyzed for pathogenicity according to records in Human Gene Mutation Database (HGMD, http://www.hgmd.cf.ac.uk/ac/index.php), ClinVar database (https://www.ncbi.nlm. nih.gov/clinvar) and the American College of Medical Genetics and Genomics (ACMG) guidelines (2) Targeted PCR was conducted according to the kit manufacturer’s protocols (2 × Master Mix KT201; Tiangen, Shanghai, China). Sanger sequencing was conducted to identify the origin of mutations (primers available on request). NM_000433.4 was used as the reference sequence for *NCF2*.

**1.3 DNA isolation, labeling, and quality control for optical genomic mapping (OGM)**

High molecular weight DNA was extracted from fresh whole blood using Bionano Prep Blood and Cell Culture DNA Isolation Kit (Bionano Genomics #30033, San Diego, CA). Optical genomic mapping and annotation of gDNA from the patients were outsourced to WeHealth Biomedical Technology Co., Ltd (Shanghai, China). DNA labeling was processed with Bionano Prep DLS DNA Labeling kit (Bionano Genomics #30071) according to the kit protocol. In brief, DNA was labeled with DLGreen fluorophores using DLE-1 enzyme at 37 °C for 2 h, digested with proteinase K at 50 °C for 30 min, and cleaned up with 1× DLE-1 buffer. Subsequently, DNA backbone was stained with DNA stain, 5× DTT, and 4× flow buffer for 1 h and homogenized overnight at 4 °C. Labeled DNA was loaded on a Saphyr chip (Bionano Genomics) and run on a Saphyr instrument (Bionano Genomics). Molecules data passed quality control (QC) are submitted for *de novo* assembly and SV analysis.

The QC criteria include: 1) half of DNA molecules should be >=150bp; 2) map rate should be >=70%; 3) effective coverage for germline variants should be >80 ×.

**1.4 *De novo* assembly and structural variant (SV) calling for OGM**

The *de novo* assembly was executed with Bionano Solve software v.3.7. Results were analyzed through SV pipeline, which compared the labeling patterns that the constructed sample genome maps and a reference using a multiple local alignment algorithm. SV events are identified when inconsistencies occur between the genome maps and the reference (Supplementary Figure S1). Reporting and direct visualization of structural variants were performed with Bionano Access software v.1.7.1. The following filtering thresholds were applied: hg38 DLE-1 SV mask was turned on (this filter is intended to mask common SV regions and highly repetitive parts of the human genome such as segmental duplications); Cutoff values of confidence for insertion/deletion = 0, inversion = 0.7, duplications = -1, intra-fusion = 0.3, inter-translocation =0.05. These confidence scores are computed based on models that were trained using simulated isolated, non-complex SVs and detected SVs from real samples. This filtering setting can also be customized. The confidence of an insertion or deletion call, which ranges from 0 to 1, reflects an estimate of the probability of the call being a true positive, or positive predictive value. It takes into account the SV size, the non-normalized p-value (log10) of the two well-aligned regions, and the non-normalized log-likelihood ratio of the poorly aligned or unaligned region. The genomic context is not explicitly taken into account; confidence scores of calls around complex regions may be less reliable.

SV calls were also filtered by comparing with an OGM dataset of 179 human population healthy control samples. Retained were only rare SVs, which were not detected in any of the population control samples. OGM also provided a distinct CNV pipeline, which allows for the detection of large unbalanced aberrations (>500 kb) based on normalized molecule coverage. This pipeline is independently and simultaneously run with SV pipeline. The default cutoff value for confidence of CNV is 0.99.

In order to optimize the number of calls per sample that would require individual and clinical interpretation, additional filtering steps can be applied. For P1 a more stringent 1) molecule count-based filtering (SV covered by molecules >10) was applied for calls obtained with the SV tool (Supplementary Figure S1); 2) overlap with a coding gene; and 3) overlap with CGD-related genes (including *CYBB*, *CYBA*, *NCF1*, *NCF2*, *NCF4*, *CYBC1*). Overlap gene was defined following the default setting provided by the manufacturer, which requires a minimum 1 bp overlap between a given SV and a gene in a 12 kb range. The gene list derives from the UCSC gene track of known canonical transcripts.

Results from both pipelines were incorporated and visualized either in a circos plot or genome browser view. The former allows for an easy overview of the detected variants at a glance (Supplementary Figure S2). The latter exhibited details of interested regions or genes (location, type, size, zygosity, …).

**Results**

**1.1 NADPH Oxidase Activity in Neutrophils of P1 was inactive**

The diagnosis of chronic granulomatous disease (CGD) was based on defective respiratory burst, a reflection of impaired NADPH oxidase activity, via DHR assay. Results were obtained for P1 and a healthy control. Gates of neutrophils were shown (Figure 1a). The PMA-stimulated peak of P1 almost overlap with the unstimulated peak with SI= 2.3 (normal range >100, Figure 1a, lower-left), and that of a healthy control (who was not related to patient) had a complete shift and with SI=191 (Figure 1b, lower-right). Therefore, NADPH oxidase activity of P1 was compromised, and she was diagnosed as CGD.

**1.2 Genetic findings on exome sequencing**

A single heterozygous variant in *NCF2* (NM_000433.4:c.1130_1135del) was identified in P1 (Figure 1b), and it was not recorded in ExAC, 1000G nor gnomAD. This variant is predicted to lead to an in-frame deletion of two amino acids (p.D377_M378del). It was designated as pathogenic according to the criteria above and to American College of Medical Genetics and Genomics (ACMG) guidelines (2). No pathogenic variants in other known CGD-related genes (including *CYBB*, *CYBA*, *NCF1*, *NCF4*, *CYBC1*) were identified in P1. Sanger sequencing of parent-child trio found this variant is maternal (Figure 1b). The in-frame deletion of D377_M378 may not lead to protein degradation but affect the PB1 domain (351 - 429aa). As known, PB1 domain is vital for interaction of NCF2 to other cytoplasmic components of NADPH oxidase (NCF4 and NCF1) (3-5). Disturbing this interaction inhibits NCF2’s translocation to membrane to assemble with CYBB and CYBA to regulate electron transfer, and thus impaired activating of superoxide production.

**References**

1. Wang S, Wang T, Xiang Q, Xiao M, Cao Y, Xu H, et al. Clinical and Molecular Features of Chronic Granulomatous Disease in Mainland China and a XL-CGD Female Infant Patient After Prenatal Diagnosis. J Clin Immunol. 2019;39(8):762-75.

2. Richards S, Aziz N, Bale S, Bick D, Das S, Gastier-Foster J, et al. Standards and guidelines for the interpretation of sequence variants: a joint consensus recommendation of the American College of Medical Genetics and Genomics and the Association for Molecular Pathology. Genet Med. 2015;17(5):405-24.

3. Wilson MI, Gill DJ, Perisic O, Quinn MT, Williams RL. PB1 domain-mediated heterodimerization in NADPH oxidase and signaling complexes of atypical protein kinase C with Par6 and p62. Mol Cell. 2003;12(1):39-50.

4. Ueyama T, Tatsuno T, Kawasaki T, Tsujibe S, Shirai Y, Sumimoto H, et al. A regulated adaptor function of p40phox: distinct p67phox membrane targeting by p40phox and by p47phox. Mol Biol Cell. 2007;18(2):441-54.

5. Honbou K, Minakami R, Yuzawa S, Takeya R, Suzuki NN, Kamakura S, et al. Full-length p40phox structure suggests a basis for regulation mechanism of its membrane binding. Embo J. 2007;26(4):1176-86.

**Figure legends**

**Figure S1. Overview of SV analysis in optical genomic mapping.**

Blue and black lines represent the DNA backbone for sample and reference. Dots on lines represents labels (CTTAAG sequence) well matched (green) and unmatched (orange) between sample and reference. SV, structural variants; OGM, optical genomic mapping; CNV, copy number variants; DGV, database of genomic variants (http://dgv.tcag.ca/dgv/app/home); UCSC, university of california santa cruz genome browser (http://genome.ucsc.edu); Green and blue bands in genome browser view represents reference and sample. *Results visulization can be exhibited in both circos plot and genome browser view. To give an example of all types of SVs detected in P1, results here were unfiltered SVs.

**Figure S2. A customized variant filtering in optical genomic mapping for P1.**

A circos plot view allows for an overview of SVs detected (locations, types and numbers). The circos plot is composed of four circles (namely, four tracks). The outmost track with number (1,2,3….X, Y) stands for simulated cytoband on each chromosome; the second track with colorful dots represents SVs (including insertion/deletion, inversion, duplication); the third track with a purple line in the middle exhibits CNVs (purple marks a baseline; outward blue and inward red indicate CNV gain and CNV loss segments, individually); The inmost track with purple circle and crossed lines shows translocation (intra- and inter- molecules).
